# Effect of glyphosate on the growth and survival of rhizobia isolated from root nodules of grass pea (*Lathyrus sativus* L.)

**DOI:** 10.1038/s41598-023-48424-7

**Published:** 2023-12-06

**Authors:** Atrsaw Asrat, Baye Sitotaw, Turki M. Dawoud, Hiba-Allah Nafidi, Mohammed Bourhia, Animut Mekuriaw, Gezahign Fentahun Wondmie

**Affiliations:** 1 Department of Biology, College of Science, Bahir Dar University, P.O. Box 79, Bahir Dar, Ethiopia; 2 Department of Botany and Microbiology, College of Science, King Saud University, P.O. Box 2455, 11451 Riyadh, Saudi Arabia; 3 Department of Food Science, Faculty of Agricultural and Food Science, Laval University, 2325, Quebec City, QC H1V OA6 Canada; 4 Department of Chemistry and Biochemistry, Faculty of Medicine and Pharmacy, Ibn Zohr University, 70000 Laayoune, Morocco

**Keywords:** Microbiology, Ecology, Environmental sciences

## Abstract

Grass pea (*L. sativus* L.) is a widely cultivated crop worldwide, forming a symbiotic relationship with nitrogen-fixing rhizobia. Glyphosate is commonly used by farmers for weed control during agricultural processes. However, the application of this chemical herbicide negatively impacts soil fertility by affecting the nitrogen-fixing rhizobia. This study aimed to assess the effects of glyphosate on rhizobia isolated from healthy and robust Grass pea plants. Specifically, Grass pea plants exhibiting vigorous growth and a healthy appearance were intentionally selected to isolate rhizobia from their root nodules. The isolated rhizobia were then characterized based on their morphological features, biochemical properties, and resistance to abiotic traits. Rhizobial isolates from grass peas exhibited Gram-negative, rod-shaped morphology, milky colony color, and variable colony sizes. Additionally, the majority displayed smooth colony surfaces on yeast extract mannitol agar medium. Based on morphological and biochemical characteristics, the isolates could be grouped under the genus Rhizobium. Optimum growth conditions for these isolates were observed at temperatures between 28 and 38 °C, pH levels ranging from 5 to 8, and salt (NaCl) concentrations of 0.5% and 1%. At a concentration of 20 mL L^−1^, glyphosate inhibited 5.52–47% of the Rhizobium population. The inhibition percentage increased to 17.1–53.38% at a concentration of 40 mL L^−1^. However, when exposed to a higher concentration (60 mL/L) of glyphosate, 87% of the isolates were inhibited. The number of colonies after glyphosate exposure was significantly dependent on concentration, and there were notable differences between treatments with varying glyphosate concentrations (*p* < 0.05). Glyphosate negatively impacted the survival of grass pea rhizobia, leading to a reduction in the Rhizobium population (CFU). However, the effect varied between *Rhizobium* isolated from grass pea root nodules.

## Introduction

Grass pea (*Lathyrus sativus* L.) is a widely cultivated food crop belonging to the Fabaceae family within the genus *Lathyrus*. It is spread worldwide and has adapted to harsh environmental conditions, including those in Ethiopia^1^. Grass pea (*L. sativus* L.) is mostly considered as an insurance crop in areas that are vulnerable to various abiotic stresses^2–4^. Additionally, it is suitable for consumption and contributes to food and nutritional security for many low-income communities^5^. Grass pea is recognized for its climate resilience, attributed to its unique ability to enhance soil fertility with low carbon emissions, hence it plays a crucial role in mitigating climate change and global warming^6^.

*L. sativus* aids in the acquisition of nutrients during the nitrogen fixation process through a symbiotic relationship with rhizobia^7^. It can grow successfully in a variety of soil types, including very poor soils and thick clay soils, because of its powerful penetrating root system. The rhizobia fix atmospheric nitrogen, which significantly contributes to greater yields for both the grass pea and subsequent crops. As a result, the crop can be grown without the use of chemical fertilizers in a long-term sustainable farming system^8^.

Herbicide chemicals have been extensively used in agricultural production to reduce and control weeds in the crop fields^9^. Limited crop land and growing population forces farmers to take all measures to increase crop production for ensuring the food security of the world^10^. Nowadays, herbicides are applied in approximately one-third of agricultural practices^11–13^. However, the environmental fate of herbicides has become a recent concern, as only a small portion of these chemicals reaches the target organisms^14, 15^. Therefore, herbicides either directly or indirectly affect the ecosystem's overall functioning^16^.

Glyphosate is one of the non-selective weed killers among pre-emergent herbicides used to control all types of undesirable plants in general, with a particular focus on herbs^17^. Larger quantities of this herbicide have been used by farmers as a pre-emergent weed control method. As a result, the primary issue arises from incorrect application, often due to sprayer failure. Herbicides should be applied correctly in the field, following the recommended concentration. The amount of glyphosate applied to the soil should be calculated based on the recommended rate and required for a specific area^18^. The hazardous component of glyphosate causes injury to rhizobia, which are responsible for nitrogen fixation in the soil environment, when it is applied at higher concentrations^19^.

This herbicide is known to cause microbial damage in the soil medium^20^. When used intensively, glyphosate can contaminate various ecosystems, negatively impacting plants, animals, and microorganisms, and ultimately leading to the degradation of food chains. The residues of glyphosate often persist in the food chain, resulting in nutritional deficiencies, particularly in vitamins and minerals, and have the potential to cause systemic toxicity^19^. The functional characterization of plants is also affected by glyphosate, as beneficial microbes play a crucial role in the production of siderophores and auxin, as well as the solubilization of phosphate and zinc uptake^19^. Glyphosate is toxic to beneficial fauna, including microflora and earthworms, and has a significant impact on soil biology.

Glyphosate herbicides enter the soil environment through direct interception of the spray by the soil surface and leaching into the soil, impacting the rhizobia population^21^. There are many reports indicating glyphosate herbicides can inhibit the number of *Rhizobium* species, although the effect varies among different species^22^. Numerous investigations have shown that non-selective glyphosate application negatively affects the nitrogen fixation process by reducing the rhizobial population^23–25^. For example, glyphosate reduced the growth rate of *Bradyrhizobium* in glyphosate-amended media and had negative effects on nodulation and N_2_-fixation in the greenhouse and field experiments^26, 27^. Consequently, the effects of glyphosate on rhizobia in various plants have been studied extensively^23, 25, 28, 29^. However, studies on the effects at different concentrations of glyphosate on *Rhizobia* isolated from grass pea (*L. sativus*) are limited. The objectives of this study were to isolate and characterize rhizobia from root nodules of grass pea (*L. sativus L),* and investigate the effect of glyphosate at different concentrations under laboratory conditions.

## Materials and methods

### Description of sampling site

**Figure 1:**
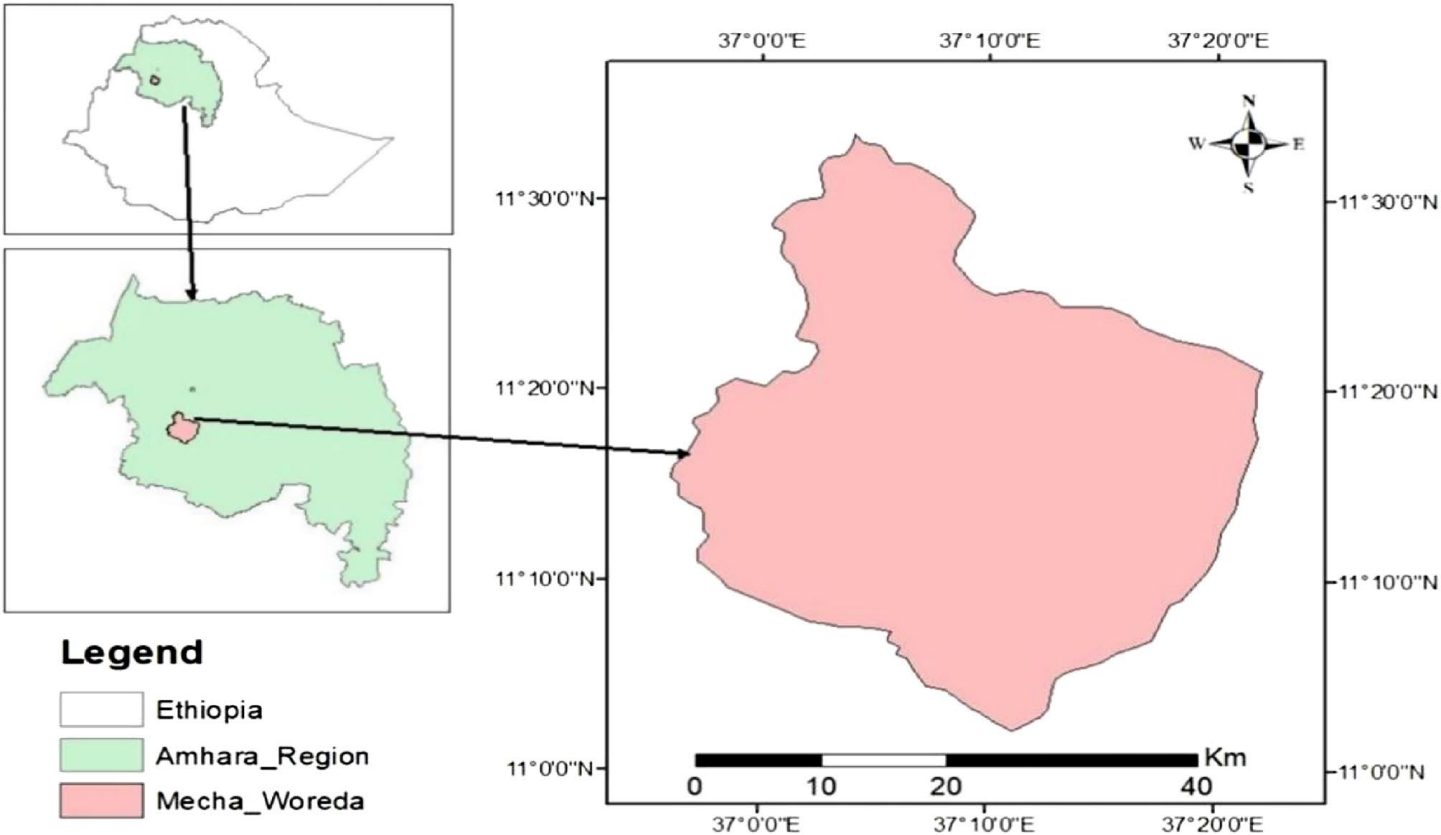
Map of study area (Alemayehu et al. 2023).

This study was conducted in the Mecha District of the West Gojjam Zone, Amhara Region, northwest Ethiopia (Fig. 1). The study area is situated at latitude and longitude ranges of 11°06′15″ to 11°38′15″ N and 36°58′44″ to 37°21′57″ E, respectively. The altitude of the district varies from 1870 to 2600 m above sea level. Mecha District is one of the major grass pea-producing areas, primarily characterized by clay soil with a pH around 4.6^30^. In this district, the most common method of weed control is the application of herbicides, particularly before plowing farmlands. According to the agricultural office of Mecha District, glyphosate application has been practiced since before 2008^31^. The mean annual rainfall and temperature are 1058 mm and 26 °C, respectively^32^.

### Sample collection

Among the 32 kebeles (the smallest administrative unit) in this district, ten kebeles, which are grass pea producing areas, were purposively selected. At each kebele, one field was randomly selected using a random sampling method. Three sampling points per field, totally 30 samples, were purposively selected at the center of each field, ensuring good growth and healthy appearance of the grass pea plants. The grass pea samples were carefully uprooted from a depth of 20–30 cm in the soil, along with their rhizospheric soil. The samples were collected aseptically, stored in an ice box, and transported to the Microbiology laboratory at the Department of Biology, Bahir Dar University. They were immediately stored at 4 °C for 48 h, after which the laboratory analyses were started.

### Isolation and characterization of Rhizobia from grass pea root Nodules

Nodules were surface disinfected using 95% alcohol for 1 min followed by 1% NaOCl for 3 min, and rinsed several times with distilled/sterile water^33^. Each nodule were crushed in normal saline solution, and a loopful of the suspension was streaked on Yeast extract Mannitol Agar medium (YEMA) constituting (g/L): Yeast extract 1, Mannitol 10, Dipotassium hydrogen phosphate 0.5, Magnesium sulfate 0.2, Sodium chloride 0.1, and Agar 15, with a pH 7.0 ± 0.2 at 25 °C (HiMedia). Inoculated plates were incubated at 28 °C for 24–48 h. Distinct isolates were picked from the plates having well separated colonies, and re-streaked to obtain a pure culture^34^.

The purified bacterial isolates were preserved at 4 °C in YEMA slants containing 0.3% (W/V) CaCO_3_^33^. The isolates were characterized on the basis of their morphology, biochemical characteristics, and physiological features (pH tolerance, temperature tolerance and salt tolerance) on YEMA^35^.

### Morphological characterization

Morphological characteristics of the isolates were determined. A loopful of 48-h broth culture (Yeast Extract Mannitol Broth (YEMB)) from each isolate was inoculated on YEMA and incubated at 28 °C for 48 h. Individual colonies were then characterized based on Gram staining, cell shape, and various colony characteristics^35, 36^.

### Gram staining

Gram staining was performed for each isolate to determine whether they were Gram-positive or Gram-negative^36^.

## Biochemical characteristics

### Congo red absorption test

It is one of the tests used to distinguish rhizobial isolates from non-rhizobial strains since *Rhizobium* species do not absorb Congo red dye. Isolates were cultured on YEMB and streaked onto YEMA-CR (Congo red) (Visakhapatnam, India) (0.5% v/v) medium to check Congo red absorption at 28 °C for 48 h^37^.

### Motility test

Bacterial isolates were inoculated into a test tube containing a SIM medium (Sigma-Aldrich, Germany) with 0.5% YEMA (Sigma-Aldrich, Germany) using sterile straight wire and incubated at 28 °C for 48 h. A positive result, indicating motility, was observed if the isolates migrated away from the line of inoculation, whereas a lack of migration indicated a negative result (non-motile)^35^.

### Indole test

Each isolate was tested for the presence of tryptophanase enzyme, which helps to hydrolyze the amino acid tryptophan. The isolates were inoculated in a test tube containing DV tryptophan broth (Sigma-Aldrich, Germany) using sterile wire loop, and incubated for 48 h at 28 °C. After incubation, 0.5 mL of Kovac’s reagent (Sigma-Aldrich, Germany) was added and mixed. The development of the red ring color indicates a positive result for indole test^35^.

### Methyl red test

The ability of isolates to perform acid fermentation were checked using MR-VP broth (contains glucose, peptone and phosphate buffers). After 48 h of incubation at 28 °C, 0.5 mL of methyl red (pH indicator) was added into the test tubes and allowed to stand for 15 min. Red color indicates a positive result for acid fermentation^35^.

### Citrate utilization test

This test was carried out to identify the bacterial isolates that use citrate as the source of carbon. The Simmons citrate agar was prepared according to the manufacturer's instructions. Then, a loopful of a 48 h bacteria culture from YEM broth was taken and incubated at 28 °C for 48 h. A change in color from green to blue indicates a positive result for citrate utilization^38^.

### Urease test

The urease test was carried out to identify the bacterial isolates that are capable of hydrolyzing urea to ammonia and carbon dioxide. In this test, the isolates were inoculated in a test tube containing 5 mL prepared urea broth (Sigma-Aldrich, Germany) and were incubated for 48 h at 28 °C. After incubation, the development of pink color was recorded as a positive result for the presence of urease^39^.

### Acid–base production test

A loopful culture from a 48-h broth of each isolate was streaked onto YEMA-BTB (Bromothymol blue) medium (Sigma-Aldrich, Germany) with a concentration of 0.25% v/v and incubated at 28 °C for 48 h. The color changes of the medium, indicative of acid/alkali production characteristics, were recorded^36^.

### Tolerance of abiotic factors

The bacterial isolates, which were refreshed in YEM Broth at 28 °C for 48 h, were inoculated onto YEMA medium. Then, they were characterized based on their responses to pH, salt, and temperature tolerance^34, 35^. Each test was performed in triplicate.

### pH tolerance

Isolates were subjected to pH tolerance test on YEMA by adjusting the pH to 4, 5, 6, 7, 8, 9 and 10 using NaOH (1N) and HCl (1N)^40^ and incubated at 28 °C for 48 h. Notably, for the pH 4 condition, the concentration of YEMA was doubled.

### Salt tolerance

The ability of isolates to tolerate salt was tested by streaking them on YEMA medium containing different salt (NaCl) concentrations (0.5%, 1%, 2%, 3%, 4%, 5%, 6%, and 7% w/v). The plates were then incubated at 28 °C for 48 h^41^.

### Temperature tolerance

Temperature tolerances of the isolates were tested by incubating them on YEM agar at 4, 10, 15, 20, 28, 38, 40, 45, and 50 °C for 48 h^42^.

### The effects of glyphosate on the rhizobial isolates

The isolates were refreshed with YEM broth, and stock solutions of glyphosate were prepared by adding 20 mL L^−1^, 40 mL L^−1^ and 60 mL L^−1^ of glyphosate through distilled Water (Mubeen et al. 2006). Three treatments (T_1_, T_2_, T_3_) with a control (without glyphosate), namely T_1_ (20 mL L^−1^), T_2_ (40 mL L^−1^), and T_3_ (60 mL L^−1^) were prepared in test tubes. Then, l mL of each filtered glyphosate was separately added to a sterilized test tube containing 10 mL YEM broth. After that, 0.1 mL of each cultured isolate was inoculated into a test tube containing the different concentrations of glyphosate, and incubated at 28 °C for 72 h. The growth of isolates in the different treatments was monitored through optical density (OD) measurement by using a UV-spectrophotometer at 600 nm. The results of the three different treatments (triplicated) were compared with glyphosate-free (control)^43^. Inhibition of glyphosate on the isolates was calculated using the formula:

$$\%PI=\frac{OD\, of\, Control -D\, of\, Treatment}{OD\, of\, Control}$$

where OD is optical density and mL L^−1^% PI is the percentage of glyphosate inhibition on the isolates^43^.

### Cell viability test

The cell viability of the isolates was evaluated in glyphosate concentrations of 20 mL L^−1^, 40 mL L^−1^, and 60 mL L^−1^ by cultivating serially diluted suspensions of 48-h-old cultures on YEM agar plates. From each treatment, 0.1 mL was transferred into YEMA medium in triplicate using the Miles drop plate method and incubated at 28 °C for 72 h for direct plate counting^44^. Additionally, a glyphosate-free culture (control) on YEM agar medium was included. Following incubation, the number of viable colonies (colony forming unit/CFU) were counted and recorded for each treatment. Each test was performed in triplicate.

### Data analysis

The data analysis was performed using SPSS version 23 statistical software packages to assess the impact of glyphosate at various concentrations on the rhizobia population. Descriptive statistics, including mean and percentages, were employed to present the values of OD and CFU for the different treatments. Furthermore, statistical differences between the treatments were analyzed using ANOVA, with means separated using Tukey’s Honestly Significant Difference (HSD) at a 95% confidence interval, and significance was set at a p-value < 0.05.

## Results

### Morphological and biochemical characteristics of rhizobia isolates

The morphological characteristics of the rhizobial isolates are summarized in Table 1. While there was considerable variation in most morphological features, colony size and texture remained relatively consistent. All isolated rhizobia from the root nodules of grass pea exhibited Gram-negative characteristics, with a rod-shaped morphology and milky-colored colonies. The majority of isolates (25 out of 30) displayed a smooth colony texture. Additionally, a significant proportion of isolates (86.6%) exhibited colony sizes ranging from 2.5 to 4.5 mm on YEMA medium (Table 1).

**Table 1:** Morphological characteristic of the Rhizobial isolates.

Isolate	Gram reaction	Shape	Colony diameter (mm)	Colony texture	Colony color
BDUs_1_	–	Rod-shaped	2.5	Smooth	Milky
BDUs_2_	–	Rod-shaped	3.0	Smooth	Milky
BDUs_3_	–	Rod-shaped	4.5	Rough	Milky
BDUs_4_	–	Rod-shaped	3.5	Smooth	Milky
BDUs_5_	–	Rod-shaped	2.0	Smooth	Milky
BDUs_6_	–	Rod-shaped	2.0	Smooth	Milky
BDUs_7_	–	Rod-shaped	2.5	Smooth	Milky
BDUs_8_	–	Rod-shaped	3.0	Smooth	Milky
BDUs_9_	–	Rod-shaped	4.0	Smooth	Milky
BDUs_10_	–	Rod-shaped	2.6	Smooth	Milky
BDUs_11_	–	Rod-shaped	3.2	Smooth	Milky
BDUs_12_	–	Rod-shaped	4.1	Smooth	Milky
BDUs_13_	–	Rod-shaped	3.5	Rough	Milky
BDUs_14_	–	Rod-shaped	2.8	Smooth	Milky
BDUs_15_	–	Rod-shaped	2.6	Smooth	Milky
BDUs_16_	–	Rod-shaped	2.0	Smooth	Milky
BDUs_17_	–	Rod-shaped	4.0	Smooth	Milky
BDUs_18_	–	Rod-shaped	3.7	Rough	Milky
BDUs_19_	–	Rod-shaped	2.0	Smooth	Milky
BDUs_20_	–	Rod-shaped	4.0	Smooth	Milky
BDUs_21_	–	Rod-shaped	4.2	Smooth	Milky
BDUs_22_	–	Rod-shaped	4.1	Smooth	Milky
BDUs_23_	–	Rod-shaped	4.5	Smooth	Milky
BDUs_24_	–	Rod-shaped	3.3	Smooth	Milky
BDUs_25_	–	Rod-shaped	2.6	Smooth	Milky
BDUs_26_	–	Rod-shaped	4.2	Smooth	Milky
BDUs_27_	–	Rod-shaped	2.9	Smooth	Milky
BDUs_28_	–	Rod-shaped	3.4	Rough	Milky
BDUs_29_	–	Rod-shaped	4.3	Smooth	Milky
BDUs_30_	–	Rod-shaped	3.9	Rough	Milky

Where, (–) = Gram negative bacteria.

All the isolates showed positive results for Methyl Red, Motility, Urease, and Acid–Base production, as indicated in Table 2. Moreover, the majority of isolates tested positive for the Citrate and Indole tests. It's noteworthy that the isolates underwent testing to confirm their identity as rhizobia and to rule out contamination with Agrobacterium, employing the Congo red technique. Throughout this study, isolates demonstrated a lack of absorption of Congo red added to the YEM broth medium, affirming their classification as rhizobia and excluding contamination with *Agrobacterium*. The YEM agar medium was enriched with BTB to identify the genus *Rhizobium*^36, 37^. Hence, considering the observed color change from deep green to yellow in YEMA-BTB and the positive results for the tested biochemical characteristics, it is indicative that all isolates likely belong to the genus *Rhizobium*.

**Table 2:** Biochemical characteristics of rhizobial isolates.

Isolate	Indole test	Citrate test	Methyl red test	Motility test	Urease test	Acid–Base production test
BDUs_1_	+	–	+	+	+	+
BDUs_2_	+	+	+	+	+	+
BDUs_3_	–	–	+	+	+	+
BDUs_4_	–	+	+	+	+	+
BDUs_5_	+	+	+	+	+	+
BDUs_6_	+	+	+	+	+	+
BDUs_7_	–	+	+	+	+	+
BDUs_8_	+	–	+	+	+	+
BDUs_9_	+	+	+	+	+	+
BDUs_10_	–	+	+	+	+	+
BDUs_11_	–	–	+	+	+	+
BDUs_12_	–	+	+	+	+	+
BDUs_13_	+	–	+	+	+	+
BDUs_14_	+	+	+	+	+	+
BDUs_15_	+	+	+	+	+	+
BDUs_16_	+	+	+	+	+	+
BDUs_17_	+	+	+	+	+	+
BDUs_18_	–	+	+	+	+	+
BDUs_19_	+	+	+	+	+	+
BDUs_20_	+	+	+	+	+	+
BDUs_21_	–	+	+	+	+	+
BDUs_22_	+	+	+	+	+	+
BDUs_23_	+	+	+	+	+	+
BDUs_24_	+	+	+	+	+	+
BDUs_25_	+	+	+	+	+	+
BDUs_26_	+	+	+	+	+	+
BDUs_27_	–	+	+	+	+	+
BDUs_28_	–	–	+	+	+	+
BDUs_29_	+	–	+	+	+	+
BDUs_30_	–	–	+	+	+	+

(+): positive result; (–): negative result.

### Tolerance of abiotic factors

#### pH tolerance of the isolates

In this study, isolates were tested for pH tolerance with variations ranging from pH 4 to 10 (Table 3). Results of this study also revealed that, all tested isolates showed growth at pH 5, 6, and 7. However, a notable portion of isolates, 70% at pH 4, 2.7% at pH 9, and 36.7% at pH 10 did not display growth under these specific pH conditions.

**Table 3:** Tolerance of rhizobia to different pH values.

Isolate	Medium in different pH						
	4	5	6	7	8	9	10
BDUs_1_	–	+	+	+	–	–	–
BDUs_2_	–	+	+	+	+	+	–
BDUs_3_	–	+	+	+	+	+	+
BDUs_4_	–	+	+	+	+	+	–
BDUs_5_	–	+	+	+	+	+	+
BDUs_6_	–	+	+	+	+	+	+
BDUs_7_	–	+	+	+	+	+	+
BDUs_8_	+	+	+	+	+	+	+
BDUs_9_	–	+	+	+	+	–	–
BDUs_10_	–	+	+	+	+	+	+
BDUs_11_	+	+	+	+	+	–	–
BDUs_12_	–	+	+	+	+	–	–
BDUs_13_	–	+	+	+	+	+	+
BDUs_14_	–	+	+	+	+	–	–
BDUs_15_	+	+	+	+	+	+	+
BDUs_16_	–	+	+	+	+	+	+
BDUs_17_	–	+	+	+	+	+	+
BDUs_18_	+	+	+	+	+	+	+
BDUs_19_	–	+	+	+	+	+	+
BDUs_20_	+	+	+	+	+	+	+
BDUs_21_	+	+	+	+	+	+	+
BDUs_22_	–	+	+	+	+	+	+
BDUs_23_	+	+	+	+	+	+	+
BDUs_24_	+	+	+	+	+	–	–
BDUs_25_	–	+	+	+	+	+	+
BDUs_26_	–	+	+	+	+	–	–
BDUs_27_	–	+	+	+	+	+	+
BDUs_28_	–	+	+	+	+	+	+
BDUs_29_	+	+	+	+	+	–	–
BDUs_30_	–	+	+	+	+	+	–

( +) = Presence of growth (–) = absence of growth.

#### Temperature tolerance of the isolates

In this study, all isolates demonstrated growth at temperatures of 20, 28, and 38 °C on YEMA medium. Additionally, a significant percentage of isolates, 66.7%, 83.3%, and 50% were able to survive at temperatures of 15, 45, and 50 °C, respectively (Table 4). However, none of the isolates exhibited growth at temperatures of 4 and 10 °C.

**Table 4:** Temperature tolerance of the isolate.

Isolate	Different temperature value							
	(4 °C)	(10 °C)	(15 °C)	(20 °C)	(28 °C)	(38 °C)	(45 °C)	(50 °C)
BDUs_1_	–	–	+	+	+	+	+	–
BDUs_2_	–	–	+	+	+	+	+	+
BDUs_3_	–	–	–	+	+	+	–	–
BDUs_4_	–	–	–	+	+	+	+	+
BDUs_5_	–	–	+	+	+	+	–	–
BDUs_6_	–	–	+	+	+	+	+	–
BDUs_7_	–	–	–	+	+	+	–	–
BDUs_8_	–	–	+	+	+	+	–	–
BDUs_9_	–	–	+	+	+	+	+	+
BDUs_10_	–	–	–	+	+	+	+	+
BDUs_11_	–	–	–	+	+	+	+	–
BDUs_12_	–	–	–	+	+	+	+	–
BDUs_13_	–	–	+	+	+	+	+	+
BDUs_14_	–	–	+	+	+	+	+	–
BDUs_15_	–	–	+	+	+	+	+	+
BDUs_16_	–	–	+	+	+	+	+	–
BDUs_17_	–	–	+	+	+	+	+	+
BDUs_18_	–	–	–	+	+	+	+	+
BDUs_19_	–	–	+	+	+	+	+	+
BDUs_20_	–	–	+	+	+	+	+	–
BDUs_21_	–	–	–	+	+	+	+	+
BDUs_22_	–	–	+	+	+	+	+	+
BDUs_23_	–	–	+	+	+	+	+	–
BDUs_24_	–	–	+	+	+	+	+	+
BDUs_25_	–	–	+	+	+	+	+	–
BDUs_26_	–	–	+	+	+	+	+	+
BDUs_27_	–	–	–	+	+	+	–	–
BDUs_28_	–	–	–	+	+	+	+	–
BDUs_29_	–	–	+	+	+	+	+	+
BDUs_30_	–	–	+	+	+	+	+	+

(+) = Presence of growth (–) = absence of growth.

#### Salt tolerance of the isolates 

**Figure 2:**
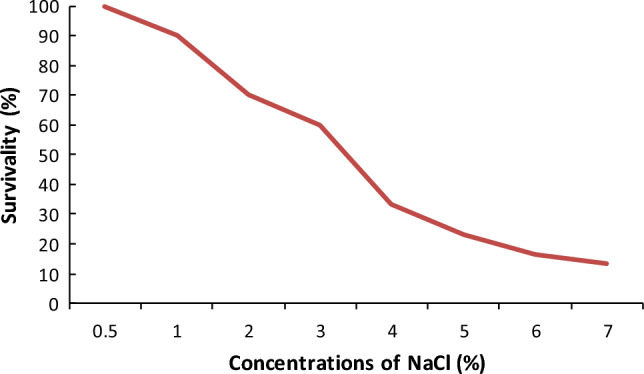
Tolerance of *Rhizobium* to different salt concentrations (W/v).

The isolates exhibited diverse responses to salt stress on YEMA medium with varying NaCl concentrations (0.5–7%). All isolates showed growth at a 0.5% NaCl concentration. However, as the salt concentration increased, the survivability of the isolates progressively declined. Specifically, some isolates did not exhibit growth beyond a 4% salt concentration, while a few isolates were able to survive at 6% and 7% salt concentrations (Table 5; Fig. 2).

**Table 5:** Growth of *Rhizobium* in the medium containing salt in different concentration (%).

Isolate	% NaCl (w/v)							
	0.5	1	2	3	4	5	6	7
BDUs_1_	+	+	+	+	–	–	–	–
BDUs_2_	+	+	+	+	+	+	+	–
BDUs_3_	+	+	+	+	–	–	–	–
BDUs_4_	+	+	–	–	–	–	–	–
BDUs_5_	+	+	+	+	+	+	+	+
BDUs_6_	+	+	+	–	–	–	–	–
BDUs_7_	+	–	–	–	–	–	–	–
BDUs_8_	+	+	+	+	+	+	+	+
BDUs_9_	+	+	+	+	+	+	+	+
BDUs_10_	+	+	–	–	–	–	–	–
BDUs_11_	+	+	–	–	–	–	–	–
BDUs_12_	+	–	–	–	–	–	–	–
BDUs_13_	+	+	+	+	+	+	+	+
BDUs_14_	+	+	+	+	–	–	–	–
BDUs_15_	+	+	+	+	–	–	–	–
BDUs_16_	+	+	+	+	+	+	–	–
BDUs_17_	+	+	+	+	+	+	–	–
BDUs_18_	+	+	–	–	–	–	–	–
BDUs_19_	+	+	+	+	–	–	–	–
BDUs_20_	+	+	+	+	–	–	–	–
BDUs_21_	+	+	–	–	–	–	–	–
BDUs_22_	+	+	+	+	+	–	–	–
BDUs_23_	+	+	+	+	+	–	–	–
BDUs_24_	+	+	+	+	+	–	–	–
BDUs_25_	+	+	+	+	–	–	–	–
BDUs_26_	+	+	+	–	–	–	–	–
BDUs_27_	+	+	–	–	–	–	–	–
BDUs_28_	+	–	–	–	–	–	–	–
BDUs_29_	+	+	+	+	–	–	–	–
BDUs_30_	+	+	+	–	–	–	–	–

(+) = Presence of growth (–) = absence of growth.

#### Effects of glyphosate on the rhizobial isolates

**Figure 3:**
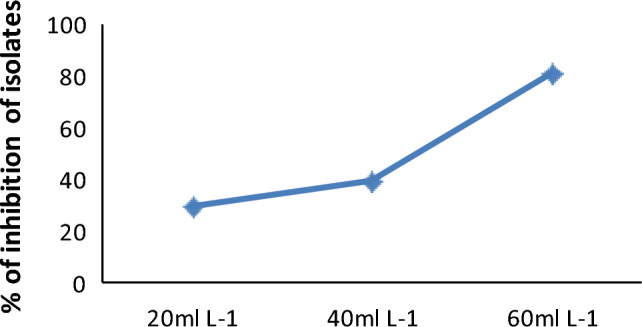
Inhibition of isolates at different concentrations of glyphosate.

The survival of Rhizobial isolates exhibited variability at different concentrations of glyphosate (Fig. 3). Nonetheless, all *Rhizobium* isolates from grass pea root nodules were cultivated in YEM broth containing different glyphosate concentrations. Among the isolates, the highest and lowest percentage of inhibition was observed at glyphosate concentrations of 60 mL L^−1^ and 20 mL L^−1^, respectively, with the isolate BDUs_7_ displaying 86.55% and 5.5% inhibition, respectively.

At the concentration of 40 mL L^−1^, BDUs_1_ and BDUs_12_ exhibited a lower percentage of inhibition (17.1%), while the isolate BDUs_7_ demonstrated the highest percentage of inhibition (53.38%). The survivability of the *Rhizobium* population at the higher concentration (60 mL L^−1^) was 14.6–24.8% and the percentage of inhibition was 75.1–85.4% respectively. *Rhizobium* isolates from grass pea root nodules did not experience complete inhibition even at higher concentrations, as evidenced by the OD values in the glyphosate-treated cultures of *Rhizobium* in YEM broth media, as indicated in Table 6.

The finding suggests that approximately 5.51–45.75% of *Rhizobium* isolates were inhibited at the recommended concentration of glyphosate (20 mL L^−1^), indicating a survival percentage within the range of 54.25–94.49% according to the company's specifications. The survivability percentage of isolates decreased with increasing concentrations of glyphosate. Specifically, about 17.13–53.38% and 7.12–85.36% of *Rhizobium* isolates were inhibited at the concentrations of 40 mL L^−1^ and 60 mL L^−1^ of liquid glyphosate, respectively (Table 6 in the supplementary file). The percentage survival of *Rhizobium* isolates ranged from 14.64 to 24.88% at 60 mL L^−1^. Following a 72-h exposure to glyphosate at this concentration, the *Rhizobium* cultures were successfully revived on YEMA medium. This finding suggests that the sensitivity of various *Rhizobium* isolates to glyphosate differs even at the same concentration.

#### Viability test

**Figure 4:**
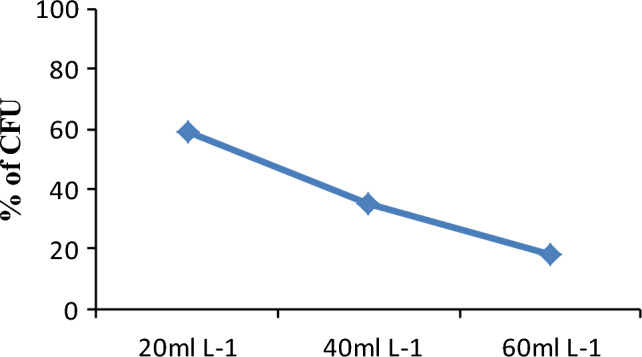
Viability of *Rhizobium* isolates (%CFU) at different concentrations of glyphosate.

The viability of the isolates was assessed by culturing serially diluted suspensions of 72-h-old cultures onto YEMA plates (Fig. 4). The percentage of colony-forming units (CFU) decreased with an increase in glyphosate concentration compared to the glyphosate-free control. Under laboratory conditions at the lowest recommended concentration of glyphosate, BDUs_30_ and BDUs_1_ exhibited the highest (92.74%) and lowest (29.29%) percentages of colony-forming units (CFU), respectively. At a concentration of 40 mL L^−1^, BDU_20_ displayed the lowest percentage of CFU (6.56%). The number of colony-forming units was found to be dependent on the concentration of glyphosate. The minimum CFU percentage (BDU_14_ and BDU_20_) and the maximum CFU percentage (BDU_30_) were 2% and 47.39%, respectively, at higher concentrations of 60 mL L^−1^ (Table 7 in the supplementary file). *Rhizobium* isolates treated with different concentrations of glyphosate were cultured on YEMA to check their viability. The growth of treated *Rhizobium* culture on YEMA medium decreased with increasing the concentration of glyphosate. At the recommended concentration of liquid glyphosate (20 mL L^−1^), 70% of the isolates demonstrated growth, with CFU percentages ranging from 51 to 83%. Conversely, 30% of the isolates exhibited growth at the same concentration, with CFU percentages ranging from 29 to 48%. In the medium containing 20 mL L^−1^, the maximum percentage of CFU was 83.33% and the minimum percentage of CFU was 29.29%. Whereas, at the concentration of 40 mL L^−1^ of glyphosate 66.66% (30–57% of CFU) and 33.33% (6–28% of CFU) of the isolates were able to grow compared to the glyphosate free (control). Maximum and minimum percentage of CFU at 40 mL L^−1^ was 69.13 and 6.59% respectively. In addition, 76.67% (10–47.39%) CFU and 23.34% (2–8%) of CFU of the isolates were able to grow at 60 mL L^−1^. The lowest percentage of CFU at 60 mL L^−1^ was 2% and the highest percentage of CFU in the same concentration was 34% of CFU compared to the control. Moreover, the percentage of CFU was reduced with the increase of concentrations, but it was not totally eliminated up to the higher concentration of liquid glyphosate (60 mL L^−1^).

## Discussion

Most of the rhizobia isolated from root nodules of grass pea did not vary in their morphological characteristics. The characteristics of native rhizobia isolates nodulating grass pea correspond to the findings reported by Kawaka et al.^45^ in Kenya: the isolates were smooth, Gram negative, and rod-shaped. Microscopic examination of this study revealed that the rhizobia isolates were rod-shaped and gram negative as similar to the findings^25, 46, 47^. Most of the isolates (86.6%) formed colonies with a diameter greater than 2.5–4.5 mm on YEMA medium, while 13.33% of the isolates exhibited a colony diameter of 2 mm. This result is consistent with the findings of Gopalakrishnan et al.^48^, who reported a colony diameter of 2–4 mm for rhizobial isolates after 5–6 days of incubation at 28 °C. 83.33% of the isolates exhibited a smooth colony appearance; however, a few isolates (BDUs_3_, BDUs_13_, BDUs_28_, BDUs_30_, and BDUs_18_) displayed a rough colony appearance. This finding aligns with the results reported by^45, 46, 49^. Colonies of rhizobia were observed on YEMA medium, and they exhibited a milky color, consistent with the results reported by Kawaka et al.^45^ in isolates from common beans.

The isolates from grass pea root nodules were tested to confirm that all isolates were rhizobia and not contaminated with *Agrobacterium*, using Congo red technique similar to the method recommended by Legesse and Assefa^50^ with isolates of rhizobia from faba bean. All of the isolates showed positive results for Methyl red, Motility, Urease, and Acid–Base production tests. In addition to this, most isolates also showed positive results for Citrate and Indole test. Results of this study indicated that rhizobial isolates were not so different biochemically since they were not varied regarding most of the tests. However, rhizobial isolates exhibited variations in their responses to carbon and nitrogen sources. This outcome aligns with the findings of Bhargava et al.^51^, who similarly demonstrated that rhizobial isolates can differ in their preferences for carbon and nitrogen sources. Biochemical and morphological characteristics of rhizobial isolates from grass pea were probably classified under the genus *Rhizobium.* The YEMA medium was enriched with BTB to identify the genus *Rhizobium* (Vincent, 1970). Therefore, the color change on YEMA-BTB was from deep green to yellow and all biochemical characteristics of rhizobial isolates suggested that all isolates could be under the genus *Rhizobium.* This was in correspondence with the findings of^50–52^.

The variation of pH in the medium might have significant effects on the growth of *Rhizobium* bacteria^19^. The fact that different strains of the same species vary widely in their pH tolerance has been reported previously^53, 54^.

In this study, 30% of the isolates demonstrated the ability to grow at pH 4. However, all isolates exhibited growth at pH levels of 5, 6, and 7. These results suggest that the tested isolates may not thrive under extremely low pH conditions or high acidity. The findings indicate that the majority of isolates were acid-tolerant, capable of surviving within the pH range of 4–7, consistent with the observations reported for the genus Rhizobium by Bhargava et al.^51^. From all isolates, 22 (73.33%) and 19 (63.33%) isolates were grown at alkaline pH 9 and 10, respectively. This was contradicted with the findings of Mekonnen^52^ on rhizobia isolated from field pea (*Pisum sativum*) that were not grown at lower pH (4.5) and at higher pH (9.5) and also contradicting with Kenasa et al.^55^ who reported that *Rhizobium* isolates were not grown at pH values lower than 4.5. In addition to this, this result was also contradicted with the findings of^56, 57^ who reported fast growing *Rhizobium* isolates appear to be more sensitive to low pH than slow growing isolates. Since, all isolates of this study were fast growing and most of them were acid-tolerant. However, it was agreed with the finding of Shetta et al.^58^ which was done on rhizobia isolated from woody legume trees grown in Saudi Arabia. In addition, this result was also similar with the findings of Kucuk et al.^59^ who reported isolates from common beans were acid-tolerant.

According to Gopalakrishnan et al.^48^, the optimal temperature for the growth of *Rhizobium* was 28–31 °C. However, the temperature range was highly strain dependent for the genus *Rhizobium.* The results of this study showed that *Rhizobium* isolates were not able to grow at 4 and 10 °C. This was disagreed with the findings of Zimmer et al.^60^ and Yuan et al.^60^ reported that isolates from legume species were able to grow at lower temperature of 10 °C. However, 50% of the isolates were survived at the temperature of 50 °C which was similar with reports of Zhang et al.^19^ and all isolates were able to grow best at the temperature 20–38 °C. However, it was contradicted with the results of Hungria et al.^61^ that were done on common beans and none of *Rhizobium* isolates grew above 38 °C. The finding was also in agreement with Kucuk et al.^59^ who reported that *Rhizobium* isolated from common beans in Turkey capable of growing above the temperature of 40 °C. This result was agreed with the findings of Mortuza et al.^62^. In addition to this, the result of this study was similar to Argaw^63^ which were done on an isolate of common bean, where 6% were survived at a temperature of 45 °C. Likewise, this result was similar with Adal^64^ on rhizobia isolated from grass peas (*L. sativus*). Moreover, the finding was corresponding to the results of Rasool et al.^65^ that was done on *Rhizobium* isolated from wild legumes and able to grow at the temperature of 30 °C, 40 °C were able to grow well while the others incubated at 50 °C showed minimal growth.

Salinity is one of the major limiting factors restricting symbiotic nitrogen fixation by *Rhizobium* species. Salt stress significantly reduces the *Rhizobium* population and affects nitrogen fixation and nodulation in legumes^66^.

In this study, a limited number of isolates exhibited high tolerance to elevated salinity. The percentage of survivability of *Rhizobium* isolates decreased with an increase in the concentration of salt. The majority of isolates (90%) demonstrated growth at 1% NaCl. However, the percentage of survival was contingent on the salt concentration, with only four isolates capable of growing at 7% salt concentration. This agreed with the results of Adal^64^ on *Rhizobium* isolated from grass pea (*Lathyrus sativus*) and some isolates surviving up to 7% of salt concentration. The finding was also agreed with the previous work of^55, 59, 67^. Salt tolerant *Rhizobium* has the potential to improve the yield of legumes under salinity stress^68^. This finding was corresponding to Zahran^69^ who reported that fast growing *Rhizobium* were able to grow well at the concentration between 0.5 and 1% of NaCl. However, this result was different from that of the findings of^50^ on *Rhizobium* strains isolated from Faba bean (*Vicia faba*) that showed that they did not grow at 0.5% NaCl concentration. In addition, this result was also different from the findings of Hewedy et al.^46^ who were done on *Rhizobium* isolated from Faba bean and reported that they were totally inhibited at 5% of NaCl and not revived in fresh medium.

This finding indicated that about (5.51–45.75%) of *Rhizobium* isolates were inhibited at the recommended concentration of glyphosate (20 mL L^−1^) and this indicates that the percentage of survival at the specification of the company was 54.25–94.49%. The result was disagreed with Drouin et al.^22^ which showed that the growth of *Rhizobium* was not affected by exposure to 20 mL L^−1^ glyphosate. In addition, this was contrasted with the reports of Mallik and Tesfai^70^ which showed that glyphosate had no effect on the growth of *Rhizobium* at concentrations up to 0.147 mL L^−1^ in YEMB. However, the initial studies by Jaworski^71^ demonstrated that 69% and 92% *R. japonicum* was inhibited by glyphosate at 0.00497 mL L^−1^ and 0.0994 mL L^−1^ concentrations respectively. The percentage of survival was decreased with increasing the concentration of glyphosate. About 17.13–53.38% and 7.12–85.36% of *Rhizobium* isolate were inhibited at the concentration 40 mL L^−1^ and 60 mL L^−1^ liquid glyphosate, respectively. The percentage survival of *Rhizobium* isolates were 14.64–24.88% at 60 mL L^−1^. This was contrasted with the results of Aynalem and Assefa^25^ reported that the percentage of survivability of *Rhizobium* was 19–22% at the concentration of 5.9 × 10^–5^ mL L^−1^. Likewise, the result of this study disagreed with the findings of Zablotowicz and Reddy^26^ reported that *R. japonicum* strains were completely inhibited at 0.497 mL L^−1^. *Rhizobium* cultures at (60 mL L^−1^) after 72 h were revived on YEMA medium. The finding indicated that the sensitivity of different *Rhizobium* isolates for glyphosate was different at the same concentration. This is similar to the findings of Dos Santos et al.^23^ that indicated commercial formulations of glyphosate herbicide were inhibited *Bradyrhizobium* and a differential response was observed in the same species.

The percentage of CFU was reduced with the increase of concentrations, but it was not totally eliminated up to the higher concentration of liquid glyphosate (60 mL L^−1^). The result of this study disagreed with the results of Aynalem and Assefa^25^ where there was no growth of bacteria after the exposure at 5.9 × 10^–5^ mL L^−1^ and 2.2 × 10^–5^ mL L^−1^ concentration of liquid glyphosate. In addition, this result was different from the findings of Zablotowicz and Reddy^26^ that showed there is complete elimination of *Rhizobium* treated with 0.497 mL L^−1^ glyphosate.

## Conclusion

Based on the current findings, it can be concluded that glyphosate herbicide application led to a reduction in the *Rhizobium* population under laboratory conditions. However, the impact varied among Rhizobium isolates. A high concentration (60 ml/L) of glyphosate resulted in a significant reduction in the *Rhizobium* population. Even at the recommended concentration (20 ml/L), there was a notable inhibition of *Rhizobium* isolates. Extreme pH levels were observed to influence the growth and survival of *Rhizobium* isolates, with a pH range of 5–8 identified as the optimum condition for the isolates. The *Rhizobium* isolates exhibited optimal growth between temperatures of 20–30 °C, and some isolates demonstrated the ability to survive up to 50 °C. However, none of the isolates were able to grow below 10 °C. High salt concentrations (4–7%) were found to impact the growth of *Rhizobium* isolates, but they demonstrated the ability to grow at lower concentrations, specifically at 0.5% and 1% NaCl. Hence, the *Rhizobium* isolates exhibited significantly varied responses to different concentrations of abiotic factors. To further understand and characterize these isolates, identification to the species level using 16S rRNA sequencing is recommended. Additionally, it is advisable to evaluate *Rhizobium* inoculum responses to different concentrations of glyphosate applications under greenhouse and field conditions for a more comprehensive understanding.

### Supplementary Information

Supplementary Information.

## Data Availability

All data generated or analysed during this study are included in this published article.
