# Supplementary material for: Effect of glyphosate on the growth and survival of rhizobia isolated from root nodules of grass pea (Lathyrus sativus L.)
Source: Sci Rep. 2023 Dec 6;13:21535. doi: 10.1038/s41598-023-48424-7 (PMC10700605; doi:10.1038/s41598-023-48424-7)
Supplement: Supplementary file 1 — Supplementary Information. [file 41598_2023_48424_MOESM1_ESM.docx]

**Appendix**

Table 6: Inhibition effects of glyphosate against *Rhizobium* at different concentration

| Isolate | | Different concentration of glyphosate | | | | | | | | | | | | | |
| --- | --- | --- | --- | --- | --- | --- | --- | --- | --- | --- | --- | --- | --- | --- | --- |
|  |  | | Control  OD | | OD at  20 mL L^-1^ | | PI (%) | | OD at  40 mL L^-1^ | | PI (%) | | OD at  60 mL L^-1^ | | PI (%) |
| BDUs_1_ | | 0.601 | | 0.529±0.019 | | 11.98 | | 0.498±0.011 | | 17.13 | | 0.147±0.008 | | 75.54 | |
| BDUs_2_ | | 0.882 | | 0.622±0.030 | | 29.47 | | 0.493±0.007 | | 44.10 | | 0.144±0.006 | | 83.67 | |
| BDUs_3_ | | 0.893 | | 0.716±0.006 | | 19.82 | | 0.517±0.016 | | 42.10 | | 0.15±0.003 | | 83.20 | |
| BDUs_4_ | | 0.943 | | 0.713±0.023 | | 24.39 | | 0.484±0.028 | | 48.67 | | 0.138±0.010 | | 85.36* | |
| BDUs_5_ | | 0.802 | | 0.682±0.022 | | 14.96 | | 0.504±0.007 | | 37.15 | | 0.149±0.004 | | 81.42 | |
| BDUs_6_ | | 0.989 | | 0.776±0.005 | | 21.53 | | 0.517±0.025 | | 47.72 | | 0.157±0.037 | | 84.12 | |
| BDUs_7_ | | 1.034 | | 0.977±0.059 | | 5.51 | | 0.482±0.022 | | 53.38 | | 0.139±0.002 | | 86.55** | |
| BDUs_8_ | | 0.913 | | 0.619±0.010 | | 32.20 | | 0.548±0.006 | | 39.97 | | 0.166±0.005 | | 81.81 | |
| BDUs_9_ | | 0.825 | | 0.562±0.008 | | 31.87 | | 0.553±0.011 | | 32.96 | | 0.154±0.005 | | 81.33 | |
| BDUs_10_ | | 0.779 | | 0.693±0.003 | | 11.04 | | 0.539±0.005 | | 30.80 | | 0.158±0.003 | | 79.71 | |
| BDUs_11_ | | 0.863 | | 0.538±0.005 | | 37.66 | | 0.484±0.005 | | 43.91 | | 0.136±0.002 | | 84.24 | |
| BDUs_12_ | | 0.601 | | 0.529±0.006 | | 12.00 | | 0.498±0.010 | | 17.13 | | 0.147±0.002 | | 75.54 | |
| BDUs_13_ | | 0.906 | | 0.635±0.005 | | 29.91 | | 0.563±0.003 | | 37.85 | | 0.174±0.005 | | 80.79 | |
| BDUs_14_ | | 0.904 | | 0.680±0.008 | | 24.77 | | 0.529±0.006 | | 41.48 | | 0.143±0.004 | | 84.18 | |
| BDUs_15_ | | 0.971 | | 0.597±0.004 | | 34.89 | | 0.481±0.004 | | 47.54 | | 0.146±0.004 | | 84.07 | |
| BDUs_16_ | | 0.643 | | 0.534±0.005 | | 16.95 | | 0.406±0.004 | | 36.85 | | 0.146±0.004 | | 77.29 | |
| BDUs_17_ | | 0.793 | | 0.563±0.006 | | 29.00 | | 0.466±0.004 | | 41.23 | | 0.136±0.002 | | 82.84 | |
| BDUs_18_ | | 0.861 | | 0.486±0.003 | | 43.55 | | 0.485±0.004 | | 43.67 | | 0.138±0.009 | | 83.97 | |
| BDUs_19_ | | 0.823 | | 0.481±0.003 | | 41.55 | | 0.465±0.004 | | 43.49 | | 0.137±0.003 | | 83.35 | |
| BDUs_20_ | | 0.867 | | 0.541±0.003 | | 37.60 | | 0.532±0.004 | | 38.63 | | 0.168±0.003 | | 80.62 | |
| BDUs_21_ | | 0.907 | | 0.512±0.003 | | 43.55 | | 0.508±0.005 | | 43.99 | | 0.169±0.003 | | 81.36 | |
| BDUs_22_ | | 0.827 | | 0.501±0.004 | | 39.41 | | 0.494±0.002 | | 40.26 | | 0.157±0.037 | | 81.01 | |
| BDUs_23_ | | 0.607 | | 0.403±0.005 | | 33.60 | | 0.367±0.003 | | 39.53 | | 0.151±0.002 | | 75.12 | |
| BDUs_24_ | | 0.793 | | 0.514±0.004 | | 35.18 | | 0.507±0.005 | | 36.06 | | 0.179±0.003 | | 77.42 | |
| BDUs_25_ | | 0.871 | | 0.544±0.004 | | 37.54 | | 0.540±0.003 | | 38.00 | | 0.210±0.013 | | 75.88 | |
| BDUs_26_ | | 0.835 | | 0.521±0.002 | | 37.60 | | 0.518±0.003 | | 37.96 | | 0.163±0.003 | | 80.47 | |
| BDUs_27_ | | 0.861 | | 0.467±0.002 | | 45.76 | | 0.462±0.004 | | 46.34 | | 0.133±0.005 | | 84.55* | |
| BDUs_28_ | | 0.823 | | 0.495±0.005 | | 39.85 | | 0.482±0.003 | | 41.43 | | 0.155±0.004 | | 81.16 | |
| BDUs_29_ | | 0.864 | | 0.544±0.004 | | 37.03 | | 0.536±0.005 | | 37.96 | | 0.202±0.001 | | 76.62 | |
| BDUs_30_ | | 0.835 | | 0.493±0.003 | | 40.95 | | 0.472±0.003 | | 43.47 | | 0.147±0.002 | | 82.39 | |

The mean difference between treatments was significant at p < 0.05. Where: OD=optical density, Control

OD = no glyphosate, PI= percentage of inhibition, BDUs= Bahir Dar University sample.

Table 7: Viability of *Rhizobium* after exposure to glyphosate

| Isolate | Control  CFU | CFU at  20 mL L^-1^ | % of  CFU | CFU at  40 mL L^-1^ | %of  CFU | CFU at  60 mL L^-1^ | %of  CFU |
| --- | --- | --- | --- | --- | --- | --- | --- |
| BDUs_1_ | 169.72 | 49.72±0.026 | 29.29 | 20.60±0.854 | 12.14 | .16±0.044 | 3.04 |
| BDUs_2_ | 259.99 | 188.27±0.056 | 72.41 | 140.16±0.060 | 54.08 | 88.66±0.072 | 34.10 |
| BDUs_3_ | 290.26 | 174.52±0.046 | 60.13 | 148.61±0.044 | 51.20 | 86.61±0.036 | 29.84 |
| BDUs_4_ | 322.88 | 179.82±0.036 | 55.69 | 142.16±0.046 | 44.03 | 81.60±0.044 | 25.27 |
| BDUs_5_ | 499.83 | 313.49±0.040 | 62.72 | 190.60±0.070 | 38.13 | 113.71±0.053 | 22.75 |
| BDUs_6_ | 431.44 | 221.88±0.044 | 51.42 | 137.27±0.052 | 31.81 | 99.88±0.066 | 23.15 |
| BDUs_7_ | 530.89 | 374.22±0.026 | 70.48 | 219.94±0.036 | 41.42 | 73.44±0.053 | 13.83 |
| BDUs_8_ | 386.75 | 163.55±0.072 | 42.28 | 123.21±0.046 | 31.85 | 62.55±0.036 | 16.17 |
| BDUs_9_ | 291.77 | 230.82±0.061 | 79.11 | 168.05±0.053 | 57.59 | 100.83±0.053 | 34.55 |
| BDUs_10_ | 223.71 | 186.44±0.072 | 83.33 | 120.77±0.046 | 53.98 | 55.88±0.053 | 24.98 |
| BDUs_11_ | 296.88 | 241.05±0.061 | 81.19 | 68.99±0.062 | 23.23 | 22.61±0.056 | 17.61 |
| BDUs_12_ | 79.27 | 43.33±0.056 | 54.66 | 27.60±0.050 | 34.82 | 11.27±0.053 | 14.22 |
| BDUs_13_ | 154.83 | 109.77±0.044 | 70.89 | 47.77±0.053 | 30.85 | 5.05±0.087 | 3.26 |
| BDUs_14_ | 249.71 | 80.21±0.036 | 53.57 | 20.93±0.061 | 8.38 | 4.74±0.078 | 2.00 |
| BDUs_15_ | 459.22 | 282.45±0.060 | 61.50 | 229.49±0.072 | 49.47 | 151.16±0.056 | 32.91 |
| BDUs_16_ | 174.55 | 124.22±0.056 | 71.16 | 66.80±0.056 | 38.28 | 21.21±0.072 | 12.15 |
| BDUs_17_ | 200.78 | 105.27±0.046 | 52.42 | 39.22±0.020 | 19.53 | 12.10±0.046 | 6.02 |
| BDUs_18_ | 287.61 | 103.88±0.044 | 36.11 | 65.44±0.046 | 22.75 | 25.27±0.070 | 8.78 |
| BDUs_19_ | 311.27 | 137.05±0.087 | 44.02 | 75.94±0.072 | 24.39 | 33.32±0.062 | 10.70 |
| BDUs_20_ | 249.21 | 104.11±0.066 | 41.77 | 16.44±0.056 | 6.59 | 4.77±0.046 | 2.00 |
| BDUs_21_ | 359.38 | 170.72±0.026 | 47.50 | 66.27±0.026 | 18.43 | 9.94±0.046 | 2.76 |
| BDUs_22_ | 303.99 | 99.61±0.056 | 32.76 | 75.43±0.053 | 24.81 | 30.60±0.056 | 10.06 |
| BDUs_23_ | 169.28 | 137.66±0.082 | 81.32 | 51.61±0.046 | 30.48 | 23.77±0.026 | 14.04 |
| BDUs_24_ | 229.44 | 145.27±0.056 | 63.31 | 94.66±0.092 | 41.25 | 51.10±0.072 | 22.27 |
| BDUs_25_ | 244.99 | 117.72±0.060 | 48.04 | 70.05±0.142 | 28.59 | 38.38±0.070 | 15.66 |
| BDUs_26_ | 290.05 | 129.83±0.044 | 44.76 | 117.66±0.046 | 40.56 | 66.33±0.053 | 22.86 |
| BDUs_27_ | 290.99 | 212.94±0.061 | 73.17 | 139.55±0.085 | 47.95 | 66.38±0.095 | 22.81 |
| BDUs_28_ | 357.49 | 183.72±0.056 | 51.39 | 127.49±0.066 | 35.66 | 82.05±0.053 | 22.95 |
| BDUs_29_ | 235.16 | 153.38±0.089 | 65.22 | 115.32±0.056 | 49.04 | 64.04±0.066 | 27.23 |
| BDUs_30_ | 279.38 | 259.11±0.075 | 92.74 | 193.16±0.053 | 69.13 | 118.49±0.062 | 47.39 |

The mean difference between each treatment was significant at p < 0.05. Where: - CFU = colony forming unit, BDUs= Bahir Dar University sample.
